# Supplementary material for: Development and validation of a nomogram risk prediction model for malignancy in dermatomyositis patients: a retrospective study
Source: PeerJ. 2021 Dec 9;9:e12626. doi: 10.7717/peerj.12626 (PMC8667746; doi:10.7717/peerj.12626)
Supplement: Supplemental Information 1 [file peerj-09-12626-s001.docx]

| **Supplement table 1** Type of cancer in dermatomyositis patients with malignant tumor | | | | | | | | |
| --- | --- | --- | --- | --- | --- | --- | --- | --- |
| **Type of cancer** | **Training cohort (n=42)** | |  | **Validation cohort (n=12)** | |  | **Total(n=54)** | |
|  | **N** | **Percent(%)** |  | **N** | **Percent(%)** |  | **N** | **Percent(%)** |
| Nasopharyngeal carcinoma | 14 | 33.3 |  | 6 | 50.0 |  | 20 | 37.0 |
| Lung cancer | 6 | 14.3 |  | 3 | 25.0 |  | 9 | 16.7 |
| Breast cancer | 6 | 14.3 |  | 1 | 8.3 |  | 7 | 13.0 |
| Cervical cancer | 4 | 9.5 |  | 1 | 8.3 |  | 5 | 9.3 |
| Colon cancer | 4 | 9.5 |  | 0 | 0.0 |  | 4 | 7.4 |
| Hematological tumors | 3 | 7.1 |  | 0 | 0.0 |  | 3 | 5.6 |
| Liver cancer | 2 | 4.8 |  | 0 | 0.0 |  | 2 | 3.7 |
| Pancreatic cancer | 1 | 2.4 |  | 0 | 0.0 |  | 1 | 1.9 |
| Thyroid cancer | 1 | 2.4 |  | 1 | 8.3 |  | 2 | 3.7 |
| Gastric cancer | 1 | 2.4 |  | 0 | 0.0 |  | 1 | 1.9 |
| Total | 42 | 100.0 |  | 12 | 100.0 |  | 54 | 100.0 |
