# Supplementary material for: Development and validation of a nomogram risk prediction model for malignancy in dermatomyositis patients: a retrospective study
Source: PeerJ. 2021 Dec 9;9:e12626. doi: 10.7717/peerj.12626 (PMC8667746; doi:10.7717/peerj.12626)
Supplement: Supplemental Information 2 [file peerj-09-12626-s002.docx]

| **Supplement table 2** Points of risk factors and risk of total points | |
| --- | --- |
| **Factor** | **Points** |
| Age |  |
| <50y | 0 |
| ≥50y | 93 |
| Interstitial lung disease |  |
| no | 75 |
| yes | 0 |
| Dysphagia |  |
| no | 0 |
| yes | 100 |
| Refractory itching |  |
| no | 0 |
| yes | 100 |
| Creatine kinase |  |
| <198U/L | 0 |
| ≥198U/L | 70 |
| **Total Points** | **Risk** |
| 45 | 0.05 |
| 97 | 0.1 |
| 154 | 0.2 |
| 192 | 0.3 |
| 223 | 0.4 |
| 252 | 0.5 |
| 281 | 0.6 |
| 312 | 0.7 |
| 350 | 0.8 |
| 407 | 0.9 |
| 460 | 0.95 |
